# Patient experience of an abstinence-based Indigenous residential treatment program in Northern Ontario: a descriptive qualitative study

**DOI:** 10.3389/frhs.2024.1387184

**Published:** 2024-12-24

**Authors:** T. N. Marsh, C. Eshakakogan, M. Spence, K. A. Morin, P. Oghene, A. Goertzen, F. Tahsin, G. Gauthier, Dean Sayers, Alan Ozawanimke, Brent Bissaillion, D. C. Marsh

**Affiliations:** ^1^ Northern Ontario School of Medicine University, Sudbury, ON, Canada; ^2^ North Shore Tribal Council, Mamaweswen, ON, Canada; ^3^ Health Sciences North Research Institute, Sudbury, ON, Canada; ^4^ ICES North, Sudbury, ON, Canada; ^5^ Batchewana First Nation, Batchewana, ON, Canada; ^6^ Sagamok First Nation, Sagamok, ON, Canada; ^7^ Serpent River First Nation, Serpent River, ON, Canada

**Keywords:** Indigenous, substance use, trauma, residential treatment, qualitative intergenerational trauma, Two-Eyed Seeing, Seeking Safety, harm reduction

## Abstract

**Background:**

Indigenous peoples with substance use disorders (SUD) and intergenerational trauma (IGT) face complex healthcare needs. Therefore understanding Indigenous patient experiences is crucial for enhancing care delivery, fostering engagement, and achieving optimal outcomes, yet few studies explore the motivations for seeking, staying in, and utilizing treatment from an Indigenous perspective. The goal of this study was to understand the patient experience with an abstinence-based treatment model in a residential treatment setting.

**Methods:**

A qualitative thematic study was conducted between April 2018 and February 2020 at Benbowpka treatment centre in Blind River, Ontario, Canada. We evaluated the results of the abstinence-based model intervention from the patient's perspective. The Client Quality Assurance Survey tool was employed to gather this data. The data underwent thematic analysis to derive meaningful insights.

**Results:**

A total of 157 patients were interviewed. The results were categorized into three parts: (1) Critical events that encouraged participants to seeking treatment; (2) Benefits experienced by participants while they were in the program; (3) Participants take-aways and priorities after completing the program. Core themes emerged in each category and each theme is sub-categorized into quadrants of the Medicine Wheel. Firstly, there was a critical juncture that significantly impacted participants' lives that motivated them to seek treatment at the Benbowpka Treatment Centre. Second, during the study, participants admission to the Benbowopka Treatment Centre, the participants benefitted from a holistic program that addresses spiritual, physical, mental and emotional aspects of healing. Third, participants identified tangible ways in which they implement the skills they gained during the program in their daily lives. Overall, study demonstrates that clients benefitted from both the program activities and the traditional healing practices.

**Conclusion:**

This research identified that SUD Indigenous residential treatment programs need to include culture, healing practices, activities and relationships that are part of the treatment process. This study found that the cultural elements and healing practices of the program were highly valued by clients.

## 1 Introduction

Indigenous peoples with substance use disorders (SUD) and intergenerational trauma (IGT) have a variety of complex healthcare needs (1). In Canda, “Indigenous” is used as an umbrella term for First Nations (status and non-status), Métis and Inuit. Indigenous populations, particularly in northern Ontario, face disproportionate challenges related SUD. For instance, Indigenous populations, suffer from significantly higher rates of drug poisoning deaths, largely due to historical injustices, including colonization and intergenerational trauma. The drug poisoning crisis has led to a seven-year decline in life expectancy among First Nations populations between 2015 and 2021 (2), in Canada, illustrating its devastating impact.

Indigenous-controlled treatment settings have emerged as pivotal spaces where the program is administered and managed by an Indigenous community, where Indigenous communities can access care tailored to their unique needs, values, and traditions. Central to the success of these settings is an understanding of Indigenous patient experiences, which not only informs the delivery of care but also empowers individuals to actively engage in their health journeys.

Several studies have examined Indigenous patient experiences within Indigenous-controlled treatment settings (3) and have undertaken evaluations aimed at assessing these experiences (4–8), resulting in a rich literature of insights. By centering Indigenous voices and perspectives, we can recognize the unique cultural, historical, and systemic factors that shape Indigenous patient experiences is essential for cultivating trust, fostering collaboration, and ultimately, achieving optimal health outcomes. However, few studies have sought to understand factors that motivate people to seek treatment, stay in treatment for longer and complete, and use the tools learned after their treatment is completed from an Indigenous lens (9–12). In 2004, Two-Eyed Seeing recognized in the academic literature when Elders presented the concept to inform an Integrative Science research project (13). The application of the concept of Two-Eyed Seeing advocates for inclusion, trust, respect, collaboration, understanding, and acceptance of the strengths that reside in both Western and Indigenous worldviews (14, 15). Two-Eyed Seeing encourages Indigenous people, health-care providers, and researchers to develop a relationship of mutual cultural respect, wherein the benefits of both worldviews are acknowledged as beneficial in the healing processes (14).

The Benbowopka Residential Treatment Center is a structured residential treatment centre for substance use disorders that services seven First Nation communities in Ontario that provides 24-h care that focuses on intensive recovery activities, such as group sessions, interactive learning about SUD, exercise, yoga, meditation, grounding, spiritual practices, and ceremony. It aims to help people with SUD become stable in their recovery before engagement in outpatient settings and before returning to an unsupervised environment, which may otherwise be detrimental to their recovery process (1).

In the realm of healthcare, substance use, trauma and treatment, the significance of culturally appropriate and community-driven options cannot be overstated, particularly within Indigenous populations. In a systematic review by Richer et al. published in 2022 that discusses the culturally tailored SUD interventions in North America, authors highlighted the need for greater investment in research focused on culturally tailored treatments across the diverse spectrum of Indigenous populations (16). Therefore, we sought to understand the patient experience of an Abstinence-Based Indigenous Residential Treatment Program in Northern Ontario to contribute to the literature on tailoring programs for Indigenous communities.

## 2 Methods

### 2.1 Design and setting

We conducted a qualitative thematic study at the Benbowopka Treatment Center in Blind River, Ontario, Canada. The Benbowopka Treatment Centre provides culturally informed addiction and mental health services to Indigenous communities within the North Shore Tribal Council (NSTC) through its role in NSTC's member First Nations. The treatment center offers programs grounded in traditional Indigenous healing practices, along with contemporary therapeutic approaches, aimed at supporting individuals struggling with substance use and related issues. The North Shore Tribal Council (NSTC) is a coalition of seven First Nations communities located along the northern shore of Lake Huron in Ontario, Canada. These communities work together to address common goals, strengthen cultural ties, and support socio-economic development while preserving traditional knowledge and values. The NSTC provides a range of services, including health, education, employment, and social services, to its member First Nations.

Elders and other cultural informants were involved in guiding the research process in the data analysis and ethics section throughout this project. Benbowopka Treatment Centre started in 1991 as a 12-Step Alcoholics Anonymous model of intervention to address alcohol addiction prominent in many First Nation communities during that time. This is part of a larger project to evaluate the effectiveness of blending Indigenous Healing Practices and Seeking Safety for the treatment of Indigenous patients with intergenerational trauma and SUD (17). However, the onset of prescription drug use on First Nation communities has shifted the need for addiction treatment towards harm reduction models of service. The shift in prevalent substances has prompted Benbowopka Treatment Centre to undergo a significant transformation, starting in 2016. The changes aimed to ensure that its services are better able to address the SUD needs of First Nations communities. The abstinence model of service that was previously being delivered at Benbowopka Treatment Centre was excluding many individuals who were seeking residential addictions treatment for prescription drug use. Individuals who were prescribed medication by their physician to address SUD or mental health challenges (such as methadone and buprenorphine/naloxone) were not eligible for admission to Benbowopka Treatment Centre because of its abstinence-based model of services. In 2014, Mamaweswen North Shore Tribal Council directed that Benbowopka Treatment Centre should move forward with a plan for the realignment of its services to a harm reduction model that would follow the best practice strategies recommended for addressing substance use disorders. Thus, this process to implement Indigenous Healing and Seeking Safety (18) treatment for Indigenous patients with a history of IGT and SUD was initiated in 2016.

This paper focuses on client satisfaction with the abstinence-based model to provide a baseline to inform the implementation of the Indigenous Healing and Seeking Safety treatment model. The implementation of the Indigenous Healing and Seeking Safety treatment model will be discussed in separate papers (18).

### 2.2 Participants

Participants included all individuals residing at the Benbowopka treatment center who completed the program between April 2018 and February 2020. All participants were adults over 18 years old. The program was available to Indigenous and non-Indigenous people. At the time of the interviews, participation in the program required participants to be abstinent from any use including opioid agonist treatment. Of 266 people in the program during that time, 183 completed the program and 157 participated in the exit survey and interviews. Demographic information was previously published in a paper by Morin et al. evaluating pre/post impacts of implementing a new program: Indigenous Health and Seeking Safety (IHSS) and changing from abstinence-based program to allowing people to be on opioid agonist treatment (OAT (19). The “pre-intervention” group in Morin et al. paper represents the population for this study. Upon starting the program, the majority were aged 18–34 (60.9%), followed by 35–54 (33.5%), 55–64 (5.3%), and fewer than 10 individuals aged 65 and over. The gender distribution was 44.4% female and 55.6% male. Of the participants, 84.2% identified as Indigenous, while 14.3% did not, and fewer than 10 individuals had unknown status. A significant portion (84.8%) resided on reserve, while 8.8% were off reserve, and 2.4% had unknown residency. The majority lived in rural areas (48.5%) and the North (83.1%). At intake, the primary substance reported was alcohol (86.0%), followed by opioids (5.4%), stimulants (6.2%), and cannabis (2.3%). 3.4% had prior opioid agonist therapy (OAT).

### 2.3 Measures and procedures

We analyzed data from satisfaction surveys and exit interviews (Supplementary Material S1), which were part of the routine program and collected by program staff to gauge clients' perspectives and satisfaction with the program. The study had not yet been conceptualized or initiated when the data collection began. Research staff obtained retrospective consent to use the previously collected data. Two methods of data collection were employed: surveys and interviews. Survey data were gathered using the Client Quality Assurance Survey tool, while interviews utilized a set of 11 in-depth questions designed to explore all aspects of the treatment experience. These questions covered reasons for entering the program, overall experience, interactions with counselors and program staff, overall satisfaction, and recommended improvements. All interviews were conducted upon discharge from the program and data was collected in writing (note taking). The data was stored by program staff according to their organization's standards. Research staff analyzed anatomized data, and identifying information was removed from the survey or interview data.

### 2.4 Data analysis

All results from the Client Quality Assurance tool which included surveys and interviews were subjected to a qualitative thematic analysis. This method was selected to be consistent with cultural data analysis models that require more involvement and interpretation from the researcher (13). The data analysis process involved several key steps to ensure rigor and transparency. In step one, a student research assistant read and re-read to identify and describe implicit and explicit ideas within the data (20) using thematic analysis, with codes developed inductively based on recurring themes. A detailed coding schema, including the types and frequencies of codes as well as their hierarchical relationships. Codes were then developed to represent the identified themes and link the raw data as summary markers. Code frequencies and code occurrences were then compared, and the emerging relationships between the codes were graphically displayed. The identified themes were then mapped onto the Medicine Wheel framework—this is described more in depth in the results section — categorizing them into its quadrants to reflect the holistic aspects of healing—spiritual, physical, mental, and emotional. Finally, in step four, emerging themes were identified (20, 21).

Triangulation was employed by having two researchers independently code the data and compare results, with any discrepancies addressed collaboratively. To mitigate researcher bias, anonymized data was made accessible to staff and researchers for independent verification, and procedures ensured that adverse findings were considered and incorporated appropriately. These themes were shared with the director and staff of Benbowpka to confirm whether they aligned with their observations and experiences, thereby enhancing the credibility of the results. Sharing the themes with program staff also provided context to ensure their relevance to the program's specific setting and helped secure staff ownership and buy-in. Additionally, their feedback was valuable in writing the discussion section of the manuscript, as they offered insights on how the themes could translate into actionable steps to improve the clients' experience.

An audit trail was maintained to track the analysis process, with regular discussions among the main researcher and Elder (defined in Table 1) to resolve disagreements through consensus. After discussion, the Elders who guided this research process explored the four core themes and confirmed that these themes connected with the teachings and four quadrants of the Medicine Wheel. The Elders recommended that the results be depicted through the lens of the Medicine Wheel to authenticate the Two-Eyed Seeing and the Indigenous decolonizing methodology (22). Although the program was for people who use substances of all kinds, in this abstinence-based model of care the Western teachings was based on many of the teachings used in Alcoholic Alcoholics Anonymous (AA) (23): an organization for people who drink too much alcohol and want to cure themselves of this habit.

**Table 1:** Definition of terms to depict indigenous health practices used in this study.

Key terms	Definition
Sweat lodge Ceremony	The Sweat Lodge is often a low, dome-shaped, structure heated by hot rocks which produce steam as water is poured on them, raising the temperature to induce heavy sweating among participants and physical and spiritual cleansing. Ceremonies often include traditional prayers and songs. It is conducted by an Elder or knowledge Carrier.
Elder	An Elder is someone with a comprehensive understanding of Indigenous Ways of Knowing and the connection to the universe, including the land, animals, seasons, ceremonies and all life.
Smudging	Smudging is a sacred ceremony of most First Nations. Depending on the geographic location, sweetgrass, sage and/or cedar can be burned to purify the body, mind, heart and spirit of all persons who enter the ceremonial area.

### 2.5 Ethics

This research was in line with the tenets of the Canadian Institutes of Health Research (2011) *Guidelines for Research Involving Aboriginal People* and the *Tri-Council Policy Statement for Ethical Conduct for Research Involving Humans* (24)*.* The study received approval from Laurentian University's Ethics Board in May 2017. The authors used a decolonizing approach to ensure that the process that was ethically and culturally acceptable in conducting research with Indigenous peoples (25, 26). To be consistent with a decolonization approach, the honoring of cultural informants and knowledge was important. Considering the study incorporated Indigenous traditional healing practices, the Elders and the entire staff team as well as Mamaweswen, North Shore Tribal Council, Indigenous scholars, and clinicians (also called cultural informants) were consulted throughout the research process. Furthermore, Two-Eyed Seeing was used to honor the strengths of both Indigenous and Western knowledge, data analysis, knowledge translation, and program development (14). Two-Eyed Seeing is an Indigenous decolonizing methodology that provided this project with an inclusive philosophical, theoretical, and methodological approach. Two-Eyed Seeing was first discussed in the literature in 2004 by Elder Albert Marshall from the Eskasoni Mik'maw Nation in Nova Scotia (22, 27).

### 2.6 Rigor and reflexivity

The inclusion of researchers with both academic backgrounds and clinical experience allowed for a balanced and reflective stance during data collection and analysis. The research team, led by the first author, who possesses qualitative doctoral training and extensive clinical experience with people with Post-traumatic Stress Disorder (PTSD) and SUD, established a rapport with participants and staff, fostering a trusting atmosphere within the groups. As an Indigenous scholar and researcher, she identified with the study participants. Although she is not a member of this cultural group, they come from a culture with a similar history of colonization, oppression, loss of land, language, and culture. Generations before her suffered multiple losses, and therefore she is aware that she has been affected by intergenerational trauma. Thus, she explicitly states that her own life experiences, biases, and personal views would influence the findings at the outset of this research. Her interest in this research topic was ignited by a passion for helping people heal from intergenerational trauma and SUD. She believes that we can heal when we receive the proper care and treatment. As a nurse and psychotherapist with experience in South Africa and Canada, she witnessed trauma, violence and their horrific consequences. She also witnessed healing and transformation and the resilience of our nations. Moreover, the credibility and confirmability of the data were reinforced through researcher triangulation during analysis.

## 3 Results

A total of 157 participants completed the Quality Assurance tool which included a survey and interview. The focus of the study was to understand the patient's perspective on an abstinence-based model of care. Three main topics were the focus of the study: (1) Critical events that encouraged participants to seek treatment; (2) Benefit experienced by participants while they were in the program; (3) Participants takeaways after completing the program. Most of the participants reported their experiences through a holistic view—incorporating their mind, body, spirit, and physical health together. Therefore, we incorporated the four quadrants of the Medicine Wheel in each theme to help interpret the data.

**Figure 1:**
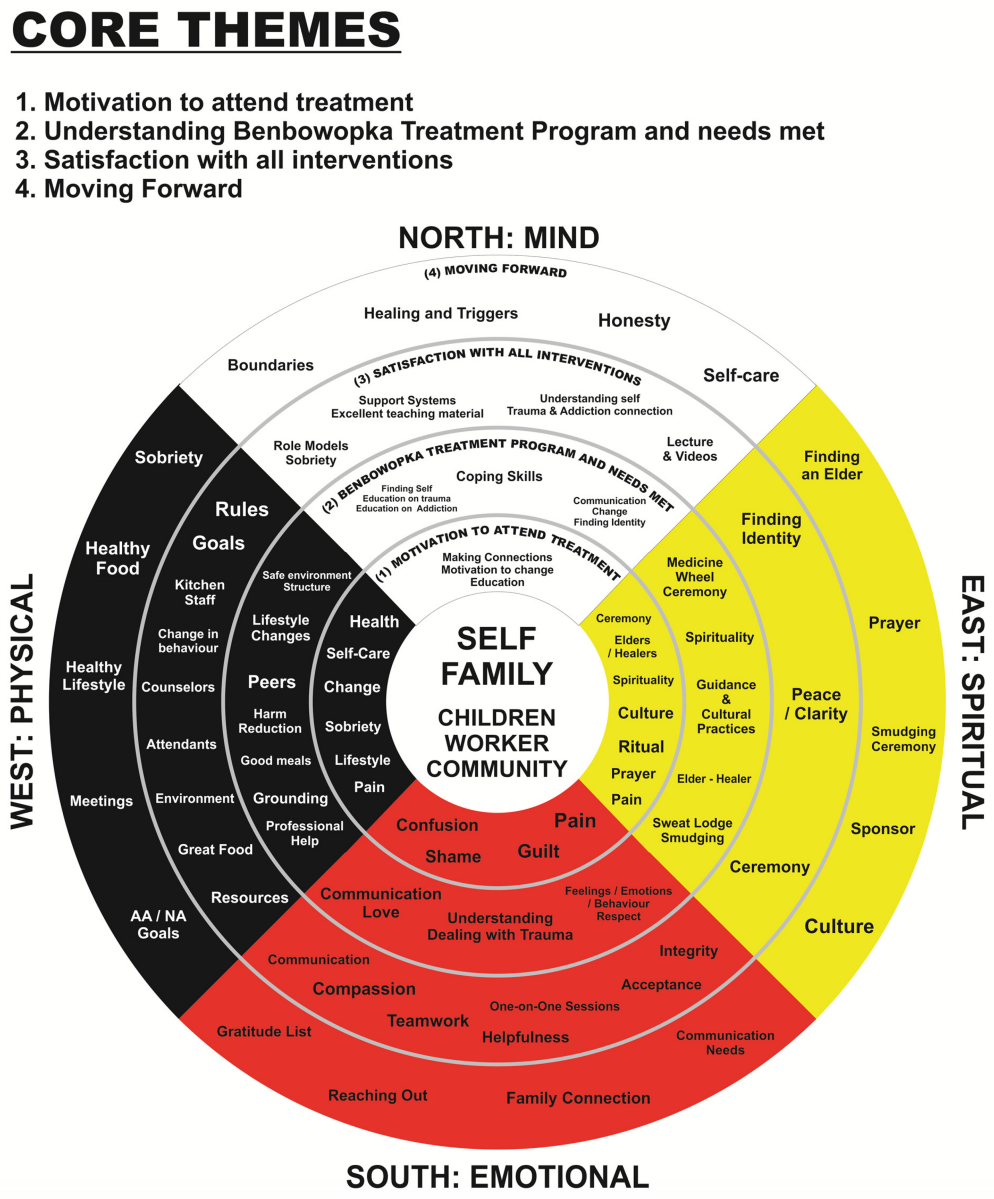
Medicine wheel—depicted in each of the three themes in the qualitative analysis.

The Medicine Wheel is depicted in Figure 1 and Table 1). The center of the Medicine Wheel represented headings (self, family, children, worker and community) or a lens, which participants used to frame their experiences. The use of the Medicine Wheel was based on an article by Rod Vickers, which was adapted from the work of Duran (28), Martin-Hill (29), and Nabigon (26). By categorizing participant perspectives within the framework of the Medicine Wheel, we gain a holistic understanding of the participants' lived experience at Benbowopka, encompassing spiritual, physical, mental, and emotional dimensions. This approach allows for a more nuanced exploration of participants' journeys. The East quadrant represents spiritual dimensions, the color is yellow; the West quadrant, embodies the physical dimension, and the color is black (26, 30); the North quadrant, symbolizes the mind the color is white (26, 31); the South quadrant, represents the emotional aspect of the person, the color is red (26, 31). The four colored quadrants of the medicine wheel represent the four directions: North, South, East, and West. The teachings begin in the East, represented by the yellow quadrant, and proceed clockwise around the circle (32)

The thematic analysis identified three main themes, each of which was further divided into subthemes corresponding to the four quadrants of the medicine wheel.

### 3.1 Critical events that encouraged participants to seek treatment

Many participants recounted pivotal moments in their lives that prompted them to seek treatment at Benbowopka. Critical events, such as life chaos, hitting rock bottom, health issues, and relationships with workers, family, work, children, and community, acted as catalysts for seeking treatment. These events can be organized into the four quadrants of the Medicine Wheel: East, West, North, and South.

#### 3.1.1 Wounded spirit and sense of self (east quadrant of the medicine wheel)

Participants described that experiences that shook their sense of purpose or connection to something greater than themselves led them to seek treatment at Benbowopka. These included moments of profound search for meaning and fulfillment through ceremony, Elders and healers, spirituality, culture, ritual, prayer, and pain. As one participant noted: “*My life became unmanageable, my children suffered, I suffered and just focused on the substance.”* Another participant reported that: “*My alcoholism was out of control, and I felt hopeless.”* Similarly, another participant said:

“I was tired of doing detox, and I was here in the program before, and I did not finish. I had to come back to finish the program. God and Creator, strangers, and my family answered my prayers. I was here before, and I needed to re-charge and become self-aware with myself and Spirituality.”

Another participant said: “I had a relapse and wanted to work on prevention and reflect on the cause and my drinking. My life became meaningless, and I did not know who I was.”

#### 3.1.2 Deteriorating physical health (west quadrant of the medicine wheel)

Participants shared narratives involving critical junctures in their health, declining self-care, need for sobriety and lifestyle change, and pain that significantly impacted their well-being and motivated them to seek support. Participants talked about the pain that they felt in their bodies due to neglect of themselves. They uttered the need to change and how they needed sobriety. Others commented about their health, how sick they felt, and how they could not even get themselves to a doctor. As one participant so eloquently stated:

“I came to gain new life skills that keep me sober and work for me, skills that pertain to cognitive behavioral therapy. The methods and treatment of this program that will help me get healthier. Becoming sober is a series of steps of other issues that will keep me alive.”

Another participant said: “My addiction and the alcohol were killing me, and I do not want to die.”

#### 3.1.3 Mental distress (north quadrant of the medicine wheel)

Participants recounted experiences related to moments of realization that spurred them to prioritize their mental well-being and seek psychological support and intervention. Many clients shared that they have lost connections with themselves, their families, their communities, and even their identities. The suffering and the pain moved them to that place and motivation where they had to find a treatment center. Many stated that they knew they needed help to understand this insanity, that they needed education about this disease. As one participant said:

“I was influenced to come here mainly because I was facing jail time, and my welfare worker advised that I come here because of my drug use, but I came here on my own free will.” Another reported: “My drinking was getting outta control, I came to better myself and get my kids back, to quit drinking, to learn more about culture.”

#### 3.1.4 Emotional challenges (south quadrant of the medicine wheel)

Finally, participants described intense emotional upheavals related to confusion, shame, pain, and guilt that deeply affected their emotional equilibrium and prompted them to seek care at Benbowpka to address their emotional wounds and find healing. Several participants mentioned their need for help, just being sick of their life, pain, and needing change. Another participant explained: “*My drinking was so out of hand that I almost died on many occasions, and that was scary. I wanted to attend the treatment program because I wanted to better my life.”*

Many of the participants shared their feelings about the loss of hope, self-esteem, identity, dignity, family, partners, and their jobs. These factors motivated them to seek help and treatment. As one participant stated:

“I needed to look at myself and regain my tools and dignity before I lost everything, even more than I already have. This treatment could help me to find my self-worth again and provided me with a whole set of tools.”

Another participant shared: “My addictions were controlling my life, taking me away from my family and my children. I feel so much guilt and shame.”

### 3.2 Benefit experienced by participants while they were in the program

The Benbowopka Treatment Centre was viewed by the participant as a holistic program that addresses spiritual, physical, mental and emotional aspects of healing.

#### 3.2.1 Benefits related to spiritual practices (eastern quadrant of the medicine wheel)

Participants reflected benefitting from spiritual including ceremonies, spiritual practices, cultural guidance, and the presence of Elders and healers. Overall, participants praised the holistic approach of Benbowopka, emphasizing its role in helping them find peace, structure, and a stronger spiritual connection. One participant stated:

“I wanted to go somewhere where I did not know anybody. I am happy I picked Benbowopka because I heard about the culture and Spirituality, I needed that. Big miigwetch (thanks) to my worker for helping me and introducing me to the program.”

Another said:

“I am so thankful for the cultural practices because I used to drink every day, and I lost touch with my culture because of my addiction. I forgot what it was like to abide by the seven grandfathers. In coming here, you guys brought me back to my roots and showed me what is important.”

They expressed gratitude for the opportunity to rediscover their identities and connect with their culture. Another participant expressed that: “*It helped me with self-respect and respecting my culture more.”*

The teachings of the Medicine Wheel and the guidance provided by staff were highly appreciated. For example, a participant explained:

“I really like all of the teachings and guidance that I received; it was so easy to get back into the culture and I want to share this with my children. Also, the program helped me build a stronger Spiritual connection.”

The Sweat Lodge Ceremony (defined in Table 1) was particularly impactful for many, offering a space for healing.

“I enjoyed the Sacred Fires, Sweats and the morning Smudges. It helped to relieve a lot of emotions, and I got a lot out of the program.” Another said: “I really liked the Sacred Fire because it helped me let go and accept the things that I needed to deal with.”

#### 3.2.2 Benefits related to the focus on emotional needs (southern quadrant of the medicine wheel)

Participants reflected benefitting from the focus on emotional needs in the program, including communication, love, understanding trauma, and managing feelings and behaviors. Many participants reflected on how powerful it was to understand how they brought substances into their lives to help them deal with the pain and suffering of the trauma and abuse. “*Yes, the counselors cared and took their time to encourage healthy risks and teach clients to bring down their guard to open up even just a little.”* They reflected on how understanding their substance use helped them feel stronger and more confident in their ability to heal. The supportive environment, fostered by staff reminders to be present and grateful, allowed participants to bond and form a supportive community. For example, one participant said: “*I understand that the program for treatment gives clients a chance to let go of baggage they are carrying while struggling with addiction. I was happy that we all received the knowledge.”* They appreciated the compassionate handling of emotions by staff members and the insights gained from the program. Participants knew they could talk during one-on-one sessions. They reported that these sessions helped them with behavioural issues, conflict and brought connection and healing. For example, one participant stated: “*The staff and counselors, they’re awesome; you can't say a bad thing about them, it's unreal; they helped us all; they never judged anyone; they just helped us.”* Another said: “*I experienced more understanding about myself; that's how much this program affected me; it helps you understand why you do what you do and gives you tools to change.”*

#### 3.2.3 Benefits related to the focus on physical aspects (western quadrant of the medicine wheel)

Overall, participants reported that their physical wellness needs were met and expressed a sense of lightness and improved self-care as a result of their time in the program. Professional help, grounding techniques, nutritious meals, peer support, harm reduction were identified as particularly helpful. They expressed feeling safe, cared for, and empowered by the supportive environment. Sharing their stories with others who had similar experiences was particularly impactful, helping them realize they were not alone and fostering a sense of camaraderie. As one participant shared:

“I am not alone; I am not the only one going through this; this program taught me that I started to use alcohol because of my trauma and that I can heal from both [the trauma and the addiction concurrently].”

They felt they were exposed to a professional team that taught them how to change their lifestyle, and all of this was given to them in a very safe environment. Hence, they reported that all their needs were met. They spoke in many ways about how they were taught to ground themselves. They expressed constant gratitude to the staff team and the kitchen staff for the great meals that they received. As one client noted: “*Yes, I feel that the program has met my needs. I have learned so much and gained so many tools. I feel so much lighter.”* And yet another said: “*They were all great at what they do here. I feel great because of them, they gave me what I needed to improve self-care.”*

#### 3.2.4 Benefits related to the focus on mental health and growth (northern quadrant of the medicine wheel)

Participants viewed the program as a psychological pathway towards leading sober and healthy lifestyles, appreciating the opportunity to learn and grow in a supportive and transformative setting. The identified aspects such as: coping skills, finding self, education on trauma and addiction, communication, change, and finding identity. Many participants mentioned that it was a safe environment, a place where they could learn more skills and begin to understand both trauma and addiction. One participant described: “*Yes, before coming here my life was unmanageable, especially my emotions. Being here, I was able to express my emotions in a healthy manner while obtaining the appropriate tools for after the completion of the program.”* Another stated: “*Alcohol/mental health treatment is a psychological and Spiritual pathway that offers tools to individuals that aspire to lead sober and healthy lifestyles.”* Others echoed: “*A place to discover yourself away from the addiction. A place to let go and a place to learn how to feel.”* Another participant said: “*You guys helped us understand all aspects of our disease. See it from all views and how it controls our lives, and you gave us tools to deal with it in a positive, healthy way.”* Other important quotes reflected included: “*The books were very helpful because answering those questions helped me identify my character defects and how the drinking affected my life.”* Another participant described that: “*I have learned some things I was doing to myself and the person I was before entering the door. I learned to let go of the pain I was carrying around for years and years.”*

### 3.3 Participants take-aways after completing the program

Participants discussed various ways in which they would continue healing after the program using the tools and knowledge they gained during the program. Their reflections are categorized in the Medicine Wheel quadrants and include: seeking guidance from Elders and sponsors, practicing gratitude and reaching out for support, setting goals for a healthier lifestyle, sobriety and focusing on healing, setting boundaries, and prioritizing self-care.

#### 3.3.1 Incorporating cultural and spiritual practices (eastern quadrant of the medicine wheel)

Participants emphasized the importance of cultural practices, such as finding an Elder and a sponsor, engaging in prayer and ceremonies (defined in Table 1), and maintaining a connection to their culture, as integral components of their journey towards sobriety and well-being. Many of the participants, as in one voice, stated that they would find an Elder and a sponsor as they continued to follow their cultural practices. Also, many talked about finding the resources that would support and help them along. As one participant pointed out: “*Going to access all available resources in Sudbury that will directly help me with my sobriety. Constantly monitor a daily healthy routine.”* Sudbury is a larger city in the near-North, the largest city centre close to the treatment program.

#### 3.3.2 Prioritizing emotional well-being (southern quadrant of the medicine wheel)

Participants highlighted the importance of gratitude, reaching out, family connection, and communication needs after they leave the program. Many expressed learning the practice of daily gratitude and reaching out for support, including utilizing AA sponsors. They also emphasized building stronger family connections and improving communication skills to express their needs effectively. For example, one participant said: “*I have learned not to be ashamed to reach out for help. I will use my AA sponsor more.”* Others claimed*: “I am confident in myself opening up. I can speak up the way I feel. I trust myself now.”* Another participant reported: “*I now have tools I can apply at home. I can share with my partner, and I can work on our communication and how to handle disagreements, and now I can sit and take a look back to see what's going on.”*

#### 3.3.3 Prioritizing physical health (western quadrant of the medicine wheel)

Participants talked about their goals, meetings, healthy lifestyle, healthy food, and sobriety after they completed the program. As in the other two quadrants, most of the participants reported the same activities, goals, and plans. Some quotes included:

“I will try my best to change, be healthier, have food in my house, take care of my house and just be kind. It’s going to be a BIG change. I am kind of scared but mostly excited. New beginnings! I will eat three meals a day, exercise more, communicate with my partner more positively, and pray more.”

Another echoed: “During my stay here at the treatment center, I have developed a comfort with the routine that I followed. I will continue this when I get back home (healthy eating, exercising, praying, smudging, chores, etc.).”

#### 3.3.4 Prioritizing mental health (northern quadrant of the medicine wheel)

Participants expressed the importance of boundaries, healing, triggers, honesty, and self-care after they complete the program. Multiple participants shared about how they healed. They discussed dealing with triggers and boundary issues. One participant described:

“I have neglected so many things that would/will make my life balanced and good quality life for myself, family and personal goals. I can and know how to use the tools and teachings I’ve learned here, and I can put them into good use.”

Another mentioned:

“I will change a lot. Attending meetings and Ceremonies. Practicing healthier thoughts and actions. I know I don’t want to drink anymore. Self-care will be the biggest thing and focusing on my children.”

## 4 Discussion

In collaboration with the North Shore Tribal Council and Benbowopka Treatment Center, this study aimed to evaluate the participants' experience of the abstinence-based treatment model. The results were categorized into three parts: (1) Critical events that encouraged participants to seeking treatment; (2) Benefit experienced by participants while they were in the program; (3) Participants take-aways and priorities after completing the program. Core themes emerged in each category and each theme is sub-categorized into quadrants of the Medicine Wheel. Firstly, there was a critical juncture that significantly impacted participants' lives that motivated them to seek treatment at the Benbowpka Treatment Centre. Second, during the participants admission to the Benbowopka Treatment Centre the participant benefitted from a holistic program that addresses spiritual, physical, mental and emotional aspects of healing. Third, participants identified tangible ways in which they implement the skills they gained during the program in their daily lives. Overall, the study demonstrate that clients benefitted from both the program activities and the traditional healing practices.

We found that participants were motivated to find their culture, identity, ritual, and Elders to help with the chaos and pain they experienced in their lives. Similarly, Smith (33) and Waldram (34) found that people who recover via traditional healings and Spirituality move faster toward healing (28, 30, 35, 36). Duran, in his seminal work with Indigenous clients concurred that traditional and cultural practices help struggling individuals with healing and letting go of substances (28) Bringing in culture and/or traditional healing into treatment has been reported as a successful element in SUD programs designed for Indigenous peoples (30, 37).

Similarly, in the Canadian context, the Round Lake Treatment Centre in British Columbia implemented a program that emphasized cultural awareness through healing circles and family involvement. Evaluation of results from this program from 1991 to 1995 indicated that most participants were no longer struggling with substance use two years after completing the program (38). Yalom and Leszez, non-indigenous practitioners shared the power of shared stories and sitting together where clients could bring and see each other's pain. The participants applauded the connections they had with others and how they were validated. This connection participants experienced with others had an even greater effect on their sense of community and moved them to hope and acceptance of their struggles (28, 35).

Many of the participants struggled with shame and guilt. Therefore, addressing patients' emotional needs or distress such as shame, guilt is vital to healing. In a scoping study, Saraiya & Lopez -Castro found substantial support for an association between shame and PTSD as well as preliminary evidence suggesting that it should be treated during trauma work (39). The finding of this study resonates with the above. Hence, the teachings about the impact of trauma, guilt, and shame need to be addressed through educational sessions, and this could be offered through the new Indigenous Healing and Seeking Safety model which addresses trauma and substance use at the same time.

The themes identified by clients regarding establishing boundaries, engaging in healing, and managing triggers are crucial in breaking these cycles of IGT and SUD. The effects of IGT experienced by previous generations impact subsequent ones, can perpetuate cycles of emotional distress and maladaptive behaviors including SUD (40–42). Establishing boundaries, engaging in healing, and managing triggers are crucial in breaking these cycles. Boundaries help protect individuals from replicating harmful patterns, while healing addresses the root causes of trauma that may have been passed down (43, 44). Recognizing and managing triggers associated with this trauma prevents re-traumatization and supports healthier responses (18).

Overall, all participants concurred that the program activities, supportive staff, and the presence of the traditional healing practices impacted their sense of safety and identity. Findings furthermore point to important in creating space for traditional Indigenous healing practices in a residential treatment center.

### 4.1 Limitations

Our analysis is based on historical and retrospective data, which comes with several limitations. One of the limitations is that the themes presented may be subjective interpretations influenced by the researchers' perspectives or biases. Therefore, the reliability and generalizability of the study's conclusions could potentially be affected by the subjective nature of thematic analysis. Additionally, the analysis might have overlooked or excluded certain aspects of the participants' recovery journeys. This exclusion could lead to a fragmented understanding of their experiences, potentially missing important nuances or perspectives that could provide a more comprehensive view of their recovery process. Thus, despite its benefits in pattern identification, the thematic analysis might not have captured the full breadth and depth of the participants’ narratives. It would have been noteworthy to have included/collaborated with the participants during the analysis phase. It is important to keep in mind that these findings were collected at a time when the program was abstinent based. Therefore, a further limitation involves the generalizability of the findings, as is the nature of qualitative work. It should be noted that the findings cannot be generalized to all residential treatment facilities in Northern Ontario, nor can this be generalized to all clients at the service. Potential bias is also important to discuss, including selection bias due to non-representative sampling, courtesy and response bias from participants potentially providing socially desirable answers. Clients who were unsatisfied with the program were likely to either drop out or choose not to complete the exit interview and survey. Additionally, observer and cultural biases may influence data interpretation, while the temporal scope of the study could affect the generalizability of findings. Careful consideration in the interpretation of results is therefore necessary. However, employing neutral and non-judgmental interview techniques and having an independent research team as well as Elders and community members helped to mitigate courtesy bias, allowing participants to express their true experiences and opinions more freely.

## 5 Conclusion

This paper has presented qualitative findings that indicate positive experiences by clients participating in a program with teachings and traditional healing practices. The results highlight the importance of incorporating Indigenous healing practices into residential treatment models to foster comprehensive and effective support for individuals recovering from substance use disorders. However, the abstinence-based model means that people using drugs do not have access to this treatment. The findings of this study will inform the subsequent implementation of a novel treatment approach for Indigenous patients with a history of IGT and SUD. Future research is encouraged to replicate this work in other Indigenous treatment centers to explore the transferability of the research findings (5, 14, 41). Additionally, longitudinal studies would be beneficial to assess the long-term effectiveness of these interventions in sustaining recovery and improving overall well-being would provide valuable insights.

## Data Availability

The datasets presented in this article are not readily available because all original data used for the purpose of this study remain under the ownership and control of the North Shore Tribal Council and can only be accessed with their permission. Requests to access the datasets should be directed to tmarsh@nosm.ca.
