# Supplementary material for: Patient experience of an abstinence-based Indigenous residential treatment program in Northern Ontario: a descriptive qualitative study
Source: Front Health Serv. 2024 Dec 24;4:1387184. doi: 10.3389/frhs.2024.1387184 (PMC11703720; doi:10.3389/frhs.2024.1387184)
Supplement: Supplementary file 1 [file Table1.docx]

Manuscript: Patient Experience of an Abstinence-Based Indigenous Residential Treatment Program in Northern Ontario: A Descriptive Qualitative Study

*Authors: Marsh TN; Eshakakogan C^,^ Spence M, Morin KA, Oghene P, Goertzen A, Tahsin F, Gauthier G, Chief Dean Sayers, Chief Alan Ozawanimke, Chief Brent Bissaillion****,*** *Marsh DC*

**Supplement 1: Survey Questions**

1. What influenced your decision to attend a treatment program?
2. Please describe your understanding of a treatment program.
3. Do you feel the treatment program meets your needs? Please explain.
4. Please rate your level of satisfaction with the cultural components - the teachings.
5. Please rate your level of satisfaction with the cultural components - the morning smudge.
6. Please rate your level of satisfaction with the cultural components - the sacred circle.
7. Please rate your level of satisfaction with the treatment program components - the lectures.
8. Please rate your level of satisfaction with the treatment program components - the counsellor interaction.
9. Please rate your level of satisfaction with the treatment program components - the one-to-one sessions.
10. Please rate your level of satisfaction with the treatment program components - the problem solving.
11. Please rate your level of satisfaction with the treatment program components - the A.A. Program.
12. Please rate your level of satisfaction with the treatment program components.
13. Which component was most helpful to you, the cultural or the programming? Please explain.
14. Please rate your level of satisfaction with assigned Residential Counsellor's responsiveness.
15. Please rate your level of satisfaction with assigned Residential Counsellor's professionalism.
16. Please rate your level of satisfaction with assigned Residential Counsellor understanding my needs.
17. Please rate your level of satisfaction with assigned Residential Counsellor's knowledge and skills.
18. Please rate your level of satisfaction with assigned Residential Counsellor's presentation of lecture/teachings?
19. Do you have any recommendations/concerns/issues for the Residential Counsellors? Please explain.
20. Please rate your level of satisfaction with Treatment Program's meals/nutrition - quantity (amount).
21. Please rate your level of satisfaction with Treatment Program's meals/nutrition - quality (freshness).
22. Please rate your level of satisfaction with Treatment Program's meals/nutrition - nutritious.
23. Do you have any recommendations/concerns/issues for the Kitchen Staff? Please explain.
24. Please rate your level of satisfaction with the Full-Time Residential Night Attendants - Ensuring House Rules are always followed.
25. Please rate your level of satisfaction with the Full-Time Residential Night Attendants - Respectful and friendly
26. Please rate your level of satisfaction with the Full-Time Residential Night attendants - Empathy and caring.
27. Please rate your level of satisfaction with the Full-Time Residential Night attendants - willingness to help a client when needed.
28. Please rate your level of satisfaction with the Full-Time Residential Night attendants - Supportive attitude.
29. Do you have any recommendations/concerns/issues for the Full-Time Residential Night Attendants? Please explain.
30. Please rate your level of satisfaction with the treatment program's consistency of the House Rules by Residential Counsellors.
31. Please rate your level of satisfaction with the treatment program's consistency of the House Rules by Full-Time Residential Night Attendants.
32. Overall rating of the level of satisfaction with the treatment program's consistency of the House Rules by all Staff.
33. What is your overall satisfaction of our treatment centre program's consistency of the House Rules between all Residential Staff? Please note any comments or concerns.
34. Did you contemplate leaving the program during your stay?
35. Did you contemplate leaving the program during your stay? If yes, during which week did you consider leaving and what influenced your decision to stay? Comments.
